# Radar UAV/Bird Trajectory Feature Classification Based on TCN-Transformer and the PC-TimeGAN Data Augmentation Framework

**DOI:** 10.3390/s26082528

**Published:** 2026-04-20

**Authors:** Fei Tong, Kun Zhang, Guisheng Liao, Lin Li, Jingwei Xu, Keting Jiang

**Affiliations:** 1 National Key Laboratory of Radar Signal Processing, Xidian University, Xi’an 710071, China; tongfei1987@xupt.edu.cn (F.T.); liaogs@xidian.edu.cn (G.L.); jwxu@xidian.edu.cn (J.X.); 2 School of Automation, Northwestern Polytechnical University, Xi’an 710129, China; linli@nwpu.edu.cn (L.L.); jiangketing@mail.nwpu.edu.cn (K.J.)

**Keywords:** low-altitude surveillance radar, UAV/bird classification, few-shot learning, TimeGAN, Transformer

## Abstract

To address the challenges of scarce unmanned aerial vehicle (UAV) track samples, severe class imbalance, and high motion similarity between UAVs and birds in low-altitude radar recognition, this paper proposes a trajectory classification method integrating a TCN-Transformer model with a physics-constrained TimeGAN (PC-TimeGAN) data augmentation framework. Specifically, the PC-TimeGAN generates high-quality, kinematically compliant UAV trajectories to alleviate data scarcity and class imbalance. A multi-scale TCN-Transformer is then constructed to comprehensively extract features, utilizing multi-kernel dilated convolutions for local temporal correlations and self-attention mechanisms for global temporal dependencies, thereby improving the discrimination between UAV and bird trajectories with similar motion patterns. Furthermore, a joint loss function combining Focal Loss and Triplet Loss is employed to optimize the decision boundaries and feature space, enhancing model robustness and generalization. Experiments on a measured dataset demonstrate that, under the 15-dimensional input setting, the proposed method achieves a UAV recall of 80.00%, an FAR of 3.15%, a precision of 64.00%, and an F1-score of 0.7111. Compared to baseline methods (e.g., SVM, LSTM, GRU, Transformer, and 1D-CNN), the proposed approach significantly improves UAV recall under limited trajectory information while keeping the false-alarm rate of misclassifying birds as UAVs low. Ultimately, this method markedly enhances the comprehensive performance of rapid track-level target classification for low-altitude surveillance radars.

## 1. Introduction

In recent years, with the rapid development of unmanned aerial vehicle (UAV) technology and intelligent networked systems, the low-altitude economy, primarily driven by UAVs and manned light aircraft, has gradually emerged as an important engine for regional economic growth and the cultivation of new-quality productive forces [1,2]. At the same time, the number and types of flying objects operating in low-altitude airspace have increased rapidly, making the airspace environment increasingly complex [3]. On the one hand, a large number of consumer-grade and industrial-grade UAVs have entered the market at relatively low cost and with low operational barriers. Their operating altitudes are generally concentrated below 1000 m, forming a vertically layered airspace structure relative to conventional civil aviation routes. On the other hand, in airport clearance zones, urban centers, and sensitive areas, incidents involving unregistered or uncertified UAVs, as well as unauthorized flights conducted without proper approval, continue to occur. Such activities have repeatedly caused airport closures, flight delays, and public safety incidents both in China and internationally, posing a serious threat to low-altitude airspace security and social stability. Recent review articles and case studies have consistently pointed out that reliable detection and identification of low-slow-small (LSS) targets in complex low-altitude environments remains one of the key challenges in low-altitude security [4].

In low-altitude scenarios, numerous natural or man-made small flying objects, such as birds, tethered balloons, and kites, are commonly present. These objects are highly similar to small UAVs in terms of size, flight altitude, speed range, and radar cross section (RCS). For low-altitude surveillance radar, it is difficult to distinguish UAVs from birds and similar targets using only simple decision rules such as velocity thresholds or RCS thresholds, which often leads to missed detections or false alarms [5]. Therefore, the classification and identification of typical aerial targets such as UAVs and birds has become a major research focus in the field of counter-UAV radar [6,7].

To address the problem of accurate classification and identification of UAVs and bird-like aerial interferers, extensive studies have been conducted by researchers both in China and abroad, yielding a series of encouraging results. Chen et al. [8] systematically analyzed the physical mechanism of the radar micro-Doppler effect and pointed out that micromotions such as UAV rotor blade rotation and bird wing flapping introduce significantly different micro-Doppler signatures into radar echoes. Building on this, Molchanov et al. [9] employed time-frequency micro-Doppler features of UAVs and birds in combination with traditional machine learning methods such as support vector machines to effectively distinguish between the two classes of targets. Furthermore, Park et al. [10] constructed a convolutional neural network (CNN)-based classification model using radar spectrograms and achieved UAV recognition based on spectral representations, demonstrating that deep learning methods can effectively mine discriminative information from radar time-frequency features. Han et al. [11] designed a CNN model based on overlaid frequency-modulated continuous-wave (FMCW) range-Doppler images for UAV detection and classification. Experimental results reported in related studies indicate that, under continuous-wave or high-resolution radar conditions, micro-Doppler-based UAV/bird classification accuracy can exceed 90% [12,13].

Some domestic researchers have also investigated target classification methods based on radar track or trajectory features [14]. In practical engineering applications, trajectory features are more general and easier to obtain, since almost all low-altitude surveillance radars output track information to higher-level command-and-control centers. Such track information typically includes batch number, timestamp, spatial position, velocity, acceleration, and heading angle. Compared with signal-level data represented by micromotion signatures, trajectory features are less sensitive to noise and impose lower requirements on hardware platform complexity. Liu, Xu, and their collaborators [5] established multidimensional motion models for UAVs and birds using a conventional surveillance radar platform, extracted statistical features such as velocity variance, track curvature, and turn rate, and then used classifiers including random forests and support vector machines for target classification, thereby verifying the feasibility of using target trajectories as discriminative features.

With the rapid development of deep learning, the application of deep neural networks to radar target classification has become an important research trend. Akyon et al. [15] constructed a UAV/bird trajectory sequence dataset and explored end-to-end processing of track sequences using models such as three-dimensional convolutional neural networks (3DCNN) [16], long short-term memory (LSTM) networks [17], and Transformer architectures [18]. Their results showed that such methods can exploit temporal contextual information to reduce UAV false detections and improve, to some extent, the discriminative capability between UAVs and birds. Jiang et al. [19] collected multidimensional target trajectory features in real-world scenarios, including azimuth, range, altitude, radial velocity, and RCS, and designed a hybrid temporal network for target classification, demonstrating that deep learning methods are more advantageous than traditional approaches based on low-dimensional motion features combined with shallow classifiers. More recently, Zhu et al. [20] proposed an inductive conformal prediction enhanced LSTM-SNN network for birds and UAVs recognition, highlighting the potential of reliability-aware deep learning models in radar target classification. In addition, Larrat et al. [21] conducted UAV classification studies using millimeter-wave radar and systematically compared multiple classification algorithms under noisy conditions, further showing that the robustness of different recognition algorithms differs significantly in complex observation environments and that the choice of classification method has an important impact on practical application performance. Several review studies [22,23,24] have further pointed out that radar-track-based deep learning methods are expected to become a key means of improving automatic target classification performance in future low-altitude surveillance radar systems.

Despite these promising advances, UAV/bird classification in real low-altitude environments still faces several major challenges. Recent survey work has also emphasized that UAV detection, classification, and tracking in complex environments still face persistent challenges in data scarcity, class imbalance, and robustness of evaluation metrics, especially in radar-based low-altitude scenarios [25]. First, methods based on micromotion features generally require radars to operate at high frequencies and with long dwell times in order to improve Doppler sensitivity. Moreover, the extraction of micromotion features places stringent demands on target signal-to-noise ratio, observation geometry, and signal processing hardware resources, making such methods difficult to deploy in long-range, cluttered, and cost-constrained low-cost low-altitude surveillance radar systems. Second, traditional methods based on trajectory features often require relatively long track sequences to extract sufficient discriminative information and reduce the risk of model underfitting. In practical applications, however, real-time target classification is highly desirable, meaning that recognition results are expected to be produced at the very beginning of track initiation. In addition, compared with bird targets, UAV trajectory samples are usually much scarcer, which easily leads to class imbalance and degrades model generalization. In essence, this is a few-shot learning problem. To address this issue, Zhou et al. [26] proposed a few-shot radar target recognition method based on multimodal feature fusion and verified the feasibility of improving radar target recognition performance through feature enhancement under few-shot conditions. Wei, Song, and their collaborators [27] used GANs to synthesize radar micro-Doppler spectrograms for augmenting datasets in tasks such as human activity classification and target recognition. Furthermore, Tang et al. [28] proposed an improved TimeGAN method for high-dimensional time-series data augmentation and demonstrated the effectiveness of such temporal generative models in downstream tasks, indicating that TimeGAN-based time-series augmentation has strong potential in complex sequential scenarios. Rahman et al. [29] employed GAN architectures under physical constraints or few-shot conditions to improve the realism of synthetic training data in terms of temporal continuity and motion consistency, thereby enhancing target classification performance in small-sample scenarios. However, most existing studies have focused on signal-level or time-frequency-domain data. For UAV/bird trajectory sequence data, which exhibit strong physical constraints and rich behavioral semantics, how to achieve rapid target classification under few-shot conditions while maintaining good model generalization under asymmetric sample distributions remains an urgent issue in current engineering practice.

Motivated by the above research background, this paper proposes a novel few-shot radar UAV/bird target classification method based on measured data and multidimensional motion features. A deep learning framework is developed by integrating a generative adversarial network for few-shot learning with temporal feature modeling, and its effectiveness is validated using measured data. Experimental results show that, when only the first five trajectory points are used for rapid recognition, the proposed method can effectively improve UAV recall while keeping the false-alarm rate within an acceptable range. The main contributions of this work are summarized as follows: (1) a physics-constrained TimeGAN (PC-TimeGAN) framework is proposed to generate physically plausible UAV trajectory samples for few-shot data augmentation; (2) a multi-scale TCN-Transformer recognition network is designed to jointly model local temporal dynamics and global trajectory dependencies for improved UAV/bird discrimination; and (3) a joint loss combining Focal Loss and Triplet Loss is adopted to improve minority-class sensitivity and feature separability under imbalanced-data conditions.

The remainder of this paper is organized as follows. Section 2 introduces the experimental platform and dataset. Section 3 presents the proposed method, including the PC-TimeGAN framework, the TCN-Transformer recognition network, and the loss function design. Section 4 reports the experimental results and analysis. Finally, Section 5 concludes the paper and discusses future work.

## 2. Experimental Platform Setup

**Figure 1:**
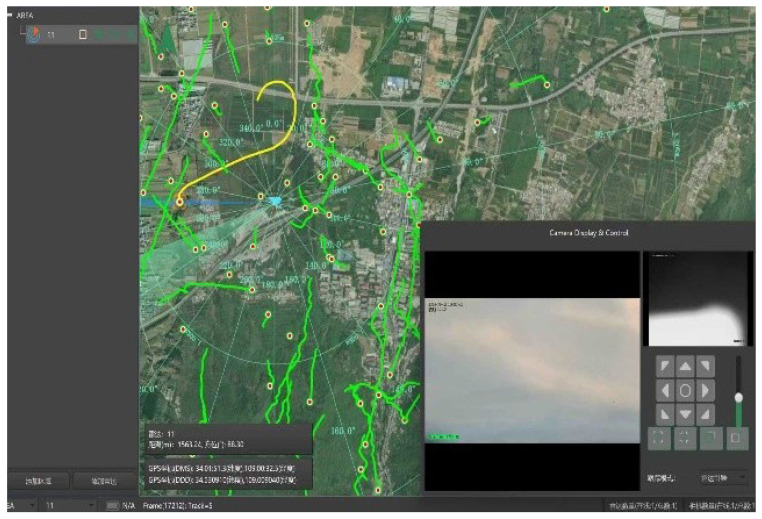
Screenshot of the terminal display of the experimental platform.

The experimental platform established in this study consisted of a military Ku-band counter-UAV radar and a dual-spectrum visible/infrared imaging system. The radar was used to detect low-altitude UAV and bird targets and to acquire their plot and track information, which served as the sample data for target classification and recognition. The dual-spectrum imaging system received radar track information and was further used for target verification, thereby providing the ground-truth target categories for labeling the radar training dataset. Due to the sensitivity of the military equipment involved, the technical specifications of both the radar and the electro-optical system cannot be disclosed in this paper. Within the range permitted for disclosure, the radar operates in the Ku band and adopts a frequency-modulated continuous-wave (FMCW) scheme, with mechanical scanning in azimuth and digital beamforming (DBF) in elevation. The elevation coverage is at least 60°, and the operating range is at least 2 km for targets with an RCS of 0.01 m^2^. The nominal angular measurement accuracy for azimuth and elevation is within 0.5°. These measurement uncertainties may propagate to the kinematic-derived features used in this study. However, since the proposed method relies on multi-frame trajectory sequences and temporal feature modeling rather than single-point measurements, the influence of instantaneous measurement errors on the final classification results is mitigated to a certain extent. In addition, Figure 1 presents a screenshot of the terminal control software to further demonstrate the authenticity of the experimental setup and data acquisition process.

## 3. Few-Shot Deep Learning Framework with GAN-Based Data Augmentation and Temporal Feature Fusion

**Figure 2:**
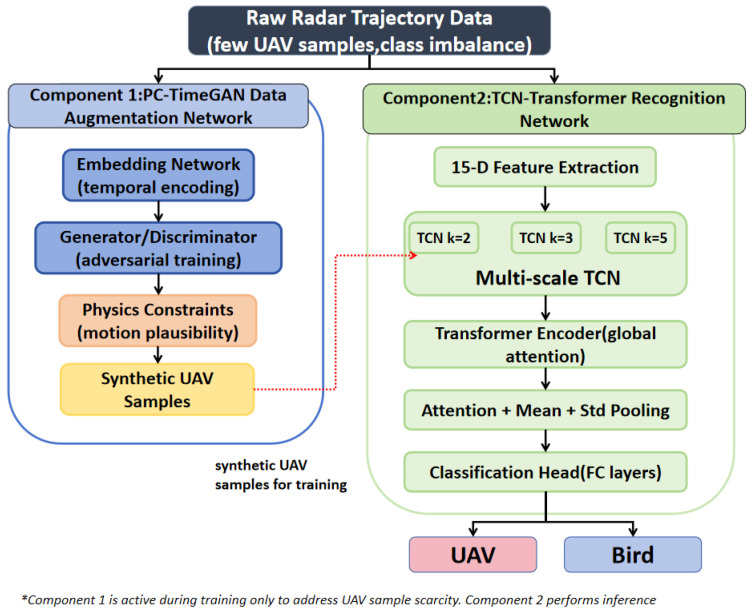
Overall Architecture of the Proposed Few-Shot Deep Learning Network.

The proposed few-shot deep learning framework in this study consists of two main components. First, a TimeGAN-based data augmentation network incorporating physical boundary constraints, referred to as the Physics-Constrained TimeGAN (PC-TimeGAN) [30], is developed to address the scarcity of UAV trajectory samples and the imbalance between UAV and bird training data. Second, a trajectory recognition network combining a multi-scale temporal convolutional network (TCN) [31] with a Transformer encoder is proposed for UAV and bird classification. The overall architecture of the proposed framework is illustrated in Figure 2.

### 3.1. Physics-Constrained TimeGAN Data Augmentation Network Design

**Figure 3:**
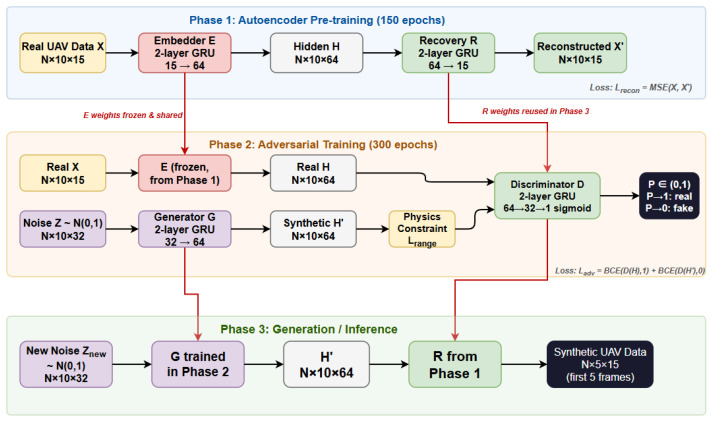
Architecture of the Physics-Constrained TimeGAN Data Augmentation Network.

TimeGAN, a specialized network model designed for time-series data generation, is capable of accurately characterizing the dynamic evolution patterns and intrinsic dependencies of temporal sequences by integrating the architectural characteristics and advantages of an autoencoder (AE) and a generative adversarial network (GAN). The model consists of three stages, as illustrated in Figure 3.

The physics-constrained strategy proposed in this study incorporates the physical boundary information of target motion into the optimization objective of the generator, thereby constraining the value range of the generated trajectories and improving the physical plausibility of the synthesized data. For synthetic samples that exceed the reasonable physical boundaries, a linear penalty term is introduced into the loss function to guide the generated trajectories to satisfy realistic kinematic constraints. The corresponding formulation is given by

(1) $$L_{\mathrm{range}}=\frac{1}{D}\sum_{d=1}^{D}\left[\max\;\left(0,\min_{d}^{\mathrm{real}}-\min_{d}^{\mathrm{fake}}\right)+\max\;\left(0,\max_{d}^{\mathrm{fake}}-\max_{d}^{\mathrm{real}}\right)\right],$$

where *d* denotes the feature dimension index, D=15 is the total number of feature dimensions, $\min_{d}^{\mathrm{real}}$ and $\max_{d}^{\mathrm{real}}$ represent the minimum and maximum values of the *d*-th dimension in the real training trajectories, respectively, and $\min_{d}^{\mathrm{fake}}$ and $\max_{d}^{\mathrm{fake}}$ denote the corresponding minimum and maximum values of the *d*-th dimension in the generated (fake) trajectories.

To further improve distributional consistency between the generated and real data, a statistical moment matching loss is introduced, which aligns the first- and second-order statistics (mean and standard deviation) of the generated feature distributions with those of the real data:

(2) $$L_{\mathrm{moment}}=\mathrm{MSE}\;\left(\mu^{\mathrm{fake}},\;\mu^{\mathrm{real}}\right)+\mathrm{MSE}\;\left(\sigma^{\mathrm{fake}},\;\sigma^{\mathrm{real}}\right),$$

where μ and σ denote the mean and standard deviation vectors computed across the batch and temporal dimensions for each feature dimension, and MSE(·,·) denotes the mean squared error.

The total loss of the generator is jointly optimized through a weighted combination of the adversarial loss, the statistical moment matching loss, and the range constraint loss, which can be expressed as

(3) $$L_{\mathrm{gen}\_\mathrm{total}}=L_{\mathrm{adv}}+\lambda_{\mathrm{moment}}L_{\mathrm{moment}}+\lambda_{\mathrm{range}}L_{\mathrm{range}},$$

where $L_{\mathrm{gen}\_\mathrm{total}}$ denotes the total loss of the generator, $L_{\mathrm{adv}}$ is the standard GAN adversarial loss implemented using binary cross-entropy, $L_{\mathrm{moment}}$ is the statistical moment matching loss, and $L_{\mathrm{range}}$ is the physical-range constraint loss. $\lambda_{\mathrm{moment}}$ and $\lambda_{\mathrm{range}}$ are the corresponding weighting coefficients used to balance the contributions of the two constraint terms. After multiple groups of comparative experiments and parameter tuning, the coefficients were set to $\lambda_{\mathrm{moment}}=10.0$ and $\lambda_{\mathrm{range}}=5.0$. This choice achieved an effective balance between adversarial learning performance and physical plausibility.

### 3.2. Trajectory Recognition Network Based on the Integration of a Multi-Scale Temporal Convolutional Network (TCN) and a Transformer Encoder

**Figure 4:**
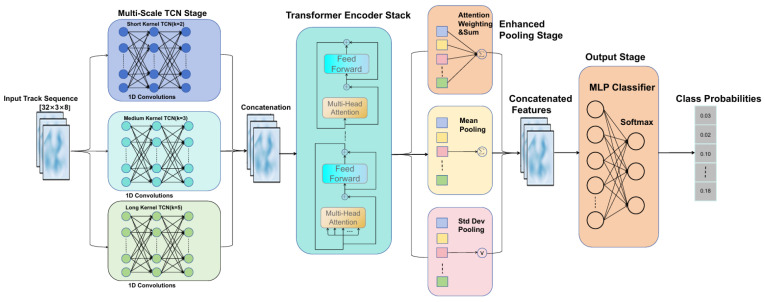
Architecture of the Trajectory Recognition Network Based on the Integration of a Multi-Scale Temporal Convolutional Network (TCN) and a Transformer Encoder.

The proposed trajectory recognition network, which integrates a multi-scale temporal convolutional network (TCN) with a Transformer encoder, consists of a front-end module and a back-end module. The overall architecture of the network is illustrated in Figure 4.

The proposed network consists of two modules:

(1) **Front-end (Multi-Scale TCN):** The front-end is composed of three parallel TCN branches with kernel sizes of 2, 3, and 5, respectively, which are designed to capture the short-term, medium-term, and long-term temporal dependencies of radar trajectory sequences. The outputs of the three branches are concatenated along the channel dimension. Based on empirical experience, the fused feature dimension is set to 64.
(2) **Back-end (Transformer):** Positional encoding is added to the features extracted by the TCN, and a Transformer encoder is then employed for global sequence modeling. The connection between the two modules is formulated as
  (4) $$X_{\mathrm{hybrid}}=\mathrm{TransformerEncoder}\left(\mathrm{TCN}\left(X\right)+PE\right),$$
  where TCN(X) denotes the output of the multi-scale TCN and PE represents the positional encoding. The resulting hybrid feature $X_{\mathrm{hybrid}}$ contains both the local features captured by the multi-scale TCN and the global dependency information modeled by the Transformer. This design provides richer and more comprehensive feature representations for subsequent UAV/bird feature learning and classification.

#### 3.2.1. Design of the Multi-Scale TCN Front-End

In trajectory recognition, target motion characteristics exhibit different patterns across different temporal scales, which can generally be categorized into short-term, medium-term, and long-term features:

- **Short-term features** (1–2 time steps): These reflect instantaneous maneuvering behaviors over a short time interval, such as target maneuverability and variations in acceleration.
- **Medium-term features** (3–5 time steps): These describe the target motion pattern over an observation window, as well as the stability of target position and velocity.
- **Long-term features** (nearly the complete trajectory): These characterize the overall motion pattern of the target and the latent class-related attributes embedded in the trajectory.

A conventional TCN designed to capture temporal dependencies at a specific scale typically adopts a single-kernel convolutional structure, which limits the model’s ability to exploit multi-scale temporal information. To enable feature extraction across different temporal scales, this study proposes a multi-scale TCN front-end architecture composed of multiple branches with substantially different convolution kernel sizes. The specific design of each branch is summarized in Table 1.

As shown in Table 1, each TCN branch is composed of two stacked TemporalBlocks. By using dilated convolutions with a dilation factor of $d=2^{l}$, the receptive field can be expanded exponentially while keeping the number of parameters relatively small. The outputs of the three branches, each with 21–22 channels, are concatenated along the channel dimension to obtain a 64-dimensional fused feature representation.

(1) TemporalBlock Structure and Dilated Convolution

The dilation factor of the *l*-th TemporalBlock is defined as

(5) $$d_{l}=2^{l},\;l=0,1,2,\ldots$$

For a single path with convolution kernel size *k* and *L* stacked layers, the overall receptive field after *L* dilated convolutions can be expressed as

(6) $$R=1+\left(k-1\right)\sum_{l=0}^{L-1}2^{l}=1+\left(k-1\right)\left(2^{L}-1\right).$$

Equation (6) is derived from the finite geometric series $\sum_{l=0}^{L-1}2^{l}=2^{L}-1$, since the dilation factor in the *l*-th TemporalBlock is defined as $d_{l}=2^{l}$. Through this strategy, the receptive field grows exponentially with network depth, enabling the model to cover longer temporal ranges while maintaining a relatively compact parameter scale.

For a single TemporalBlock, the operation can be expressed as

(7) $$f\left(x\right)=\mathrm{Dropout}\left(\mathrm{ReLU}({\mathrm{Conv}}_{2}\left(\mathrm{Dropout}\left(\mathrm{ReLU}\left({\mathrm{Conv}}_{1}\left(x\right)\right)\right)\right))\right),$$

(8) y=ReLU(f(x)+R(x)),

where ${\mathrm{Conv}}_{1}$ and ${\mathrm{Conv}}_{2}$ denote one-dimensional convolutions with dilation, and R(x) represents the residual branch. When the input and output channel dimensions are inconsistent, a 1×1 convolution is applied in the residual branch for dimension alignment. In addition, weight normalization and dropout are adopted to stabilize the training process and improve the generalization capability of the network.

Moreover, a Chomp1d module is introduced to remove redundant padding generated during convolution, ensuring that the temporal length of the output remains identical to that of the input, which facilitates residual connections and multi-layer stacking.

(2) Multi-Scale Feature Fusion and Channel Allocation

Based on prior engineering experience, the input feature dimension is set to $d_{\mathrm{in}}=15$, and the overall hidden dimension is set to $d_{\mathrm{hid}}=64$. The output channels of the three branches are approximately evenly allocated such that their total equals $d_{\mathrm{hid}}$. Specifically, the channel configuration is defined as

(9) $$d_{\mathrm{short}}\approx 21,\;d_{\mathrm{mid}}\approx 21,\;d_{\mathrm{long}}\approx 22,$$

(10) $$d_{\mathrm{short}}+d_{\mathrm{mid}}+d_{\mathrm{long}}=64.$$

The outputs of the three TCN branches are concatenated along the channel dimension to form the multi-scale temporal representation:

(11) $$X_{\mathrm{short}},\;X_{\mathrm{mid}},\;X_{\mathrm{long}}\in R^{T\times d},$$

(12) $$X_{\mathrm{TCN}}=\mathrm{Concat}\left(X_{\mathrm{short}},X_{\mathrm{mid}},X_{\mathrm{long}}\right)\in R^{T\times 64}.$$

This encoding simultaneously captures the target’s short-term maneuvering behavior, medium-term motion pattern, and long-term movement trend, thereby providing a rich feature basis for subsequent global dependency modeling using the Transformer.

#### 3.2.2. Global Dependency Modeling Based on the Transformer

(1) Positional Encoding

Since the Transformer architecture itself does not inherently encode temporal order information, positional encoding is introduced to provide the model with information about the temporal positions of sequence elements. In this study, the standard sinusoidal positional encoding is adopted, which is defined as follows:

(13) $$PE\left(\mathrm{pos},2i\right)=\sin\left(\frac{\mathrm{pos}}{10000^{2i/d_{\mathrm{model}}}}\right),$$

(14) $$PE\left(\mathrm{pos},2i+1\right)=\cos\left(\frac{\mathrm{pos}}{10000^{\left(2i+1\right)/d_{\mathrm{model}}}}\right),$$

where pos denotes the time-step position, *i* is the dimension index, and $d_{\mathrm{model}}=64$. This encoding scheme introduces no additional trainable parameters and can provide stable temporal position information for the model. The sinusoidal positional encoding is adopted following Vaswani et al. [18].

(2) Multi-Head Self-Attention Mechanism

The Transformer employs a multi-head self-attention mechanism to effectively model the dependencies between any two time steps in the sequence. In this work, four attention heads are used (num_heads=4), and the dimension of each head is given by

(15) $$d_{k}=\frac{64}{4}=16.$$

The multi-head attention operation can be formulated as

(16) $$\mathrm{MultiHead}\left(Q,K,V\right)=\mathrm{Concat}\left({\mathrm{head}}_{1},\ldots ,{\mathrm{head}}_{4}\right)W^{O},$$

where

(17) $${\mathrm{head}}_{i}=\mathrm{Attention}\left(QW_{i}^{Q},KW_{i}^{K},VW_{i}^{V}\right).$$

This structure enables the model to capture sequence dependencies in parallel across multiple subspaces, thereby substantially enhancing the comprehensiveness of temporal modeling and the expressive power of feature representation. At the same time, the use of four attention heads achieves a good balance between computational complexity and representational capacity, providing important support for model performance optimization.

(3) Feed-Forward Network (FFN)

Each Transformer layer contains a position-wise feed-forward network, which can be expressed as

(18) $$\mathrm{FFN}\left(x\right)=\mathrm{GELU}\left(xW_{1}+b_{1}\right)W_{2}+b_{2},$$

where the GELU activation function is adopted in this study.

The dimensional configuration of the feed-forward network is set to 64→256→64, following the standard Transformer design paradigm in which the hidden-layer dimension is defined as $d_{\mathrm{model}}\times 4$. The FFN is intended to perform nonlinear transformation on the feature representation at each time step, thereby further enhancing the model’s representational capability and nonlinear fitting capacity. The introduction of the Transformer encoder is particularly important for the present UAV/Bird classification task. Although the two target classes may exhibit similar local motion fragments in the early stage of a trajectory, their overall temporal evolution and cross-step dependency patterns are often different. The multi-scale TCN mainly captures local temporal variations within different receptive fields, whereas the Transformer encoder models global interactions among all time steps through multi-head self-attention. In this way, the network can adaptively emphasize more informative trajectory points and exploit nonlocal sequence dependencies, thereby improving the discriminability and robustness of the learned trajectory representations.

### 3.3. Loss Function Design

In this study, a joint loss function is designed to balance the contributions of Focal Loss and Triplet Loss through a weighting coefficient, thereby leveraging the complementary advantages of the two loss functions. It can be formulated as

(19) $$L_{\mathrm{Combined}}=\lambda L_{\mathrm{Triplet}}+L_{\mathrm{Focal}},$$

where λ is a balancing coefficient used to control the contribution of the metric learning objective to the overall loss.

**Figure 5:**
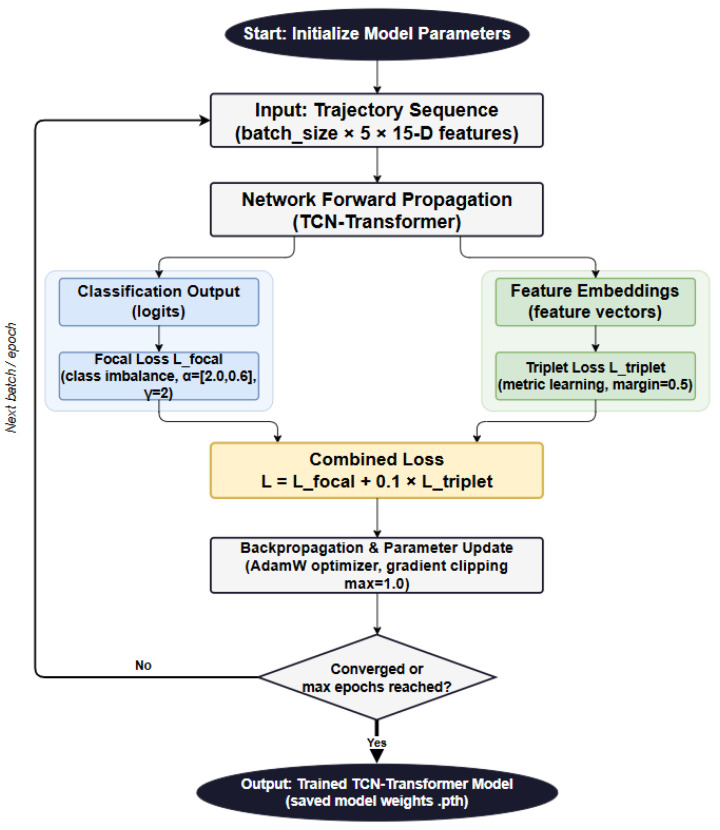
Flowchart of the Joint Loss Function Computation.

The computation process of the joint loss function is illustrated in Figure 5. The input data and network outputs are divided into two branches. One branch uses the classification logits as input to the Focal Loss in order to address the class imbalance problem, while the other branch uses the extracted feature vectors as input to the Triplet Loss to perform feature learning and metric learning. The two losses are then combined into a unified loss function, and the network parameters are optimized through backpropagation. The coefficient settings were chosen based on commonly used values in the literature and a small-scale validation comparison. Specifically, for Focal Loss, γ=2 was adopted following the empirical setting reported by Lin et al. [32], while the class weighting factors were set to $\alpha_{\mathrm{UAV}}=2.0$ and $\alpha_{\mathrm{Bird}}=0.6$ according to the imbalance ratio in the training set. For Triplet Loss, the weight was set to λ=0.1, since this value provided a better balance than λ=0, improving UAV recall by about 1–2 percentage points without noticeably increasing the false alarm rate. The Triplet margin was set to 0.5, which is a commonly used setting in metric learning.

## 4. Experimental Validation

### 4.1. Experimental Data Analysis

#### 4.1.1. Data Preprocessing

**Figure 6:**
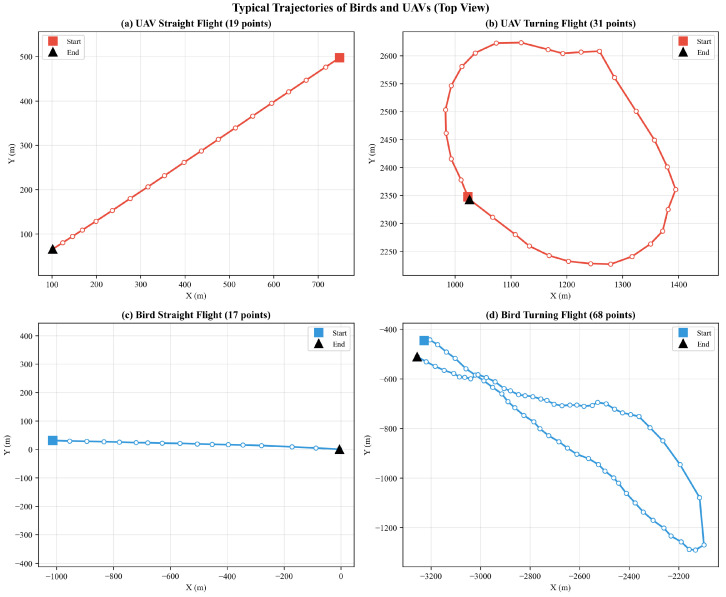
Typical Bird and UAV Trajectories.

**Figure 7:**
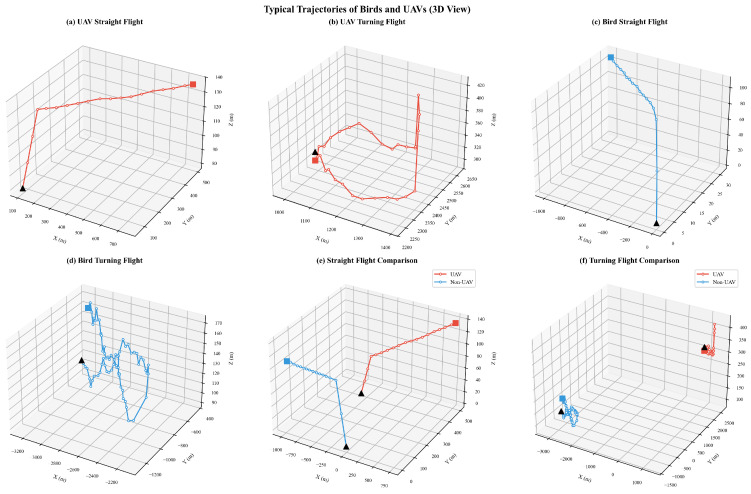
Typical Bird and UAV Trajectories (3D View).

The dataset used in this study was collected from a military radar system. The targets included in the dataset cover two categories, namely UAV and Bird targets. The UAV samples were acquired through cooperative flight tests and involved multiple UAV platforms, including the DJI Air 3, Mavic, and Mini 4 (SZ DJI Technology Co., Ltd., Shenzhen, China), and Yuneec series (Yuneec International, Kunshan, China). Various flight scenarios were designed, including constant-speed straight flight, circling, sharp turning, hovering, and vertical or lateral oscillatory maneuvers, in order to comprehensively simulate the motion characteristics of UAVs under complex mission conditions. Due to the sensitivity of the military equipment involved, the dataset used in this study cannot be made publicly available. Nevertheless, its main characteristics are described in detail in tabular and graphical form, as shown in Table 2 and Figure 6 and Figure 7.

The dataset contains a total of 9949 valid trajectory samples, including 9338 samples in the training set and 611 samples in the test set. The statistical distribution of the dataset split is summarized in Table 2. The training/test split was predefined by the supervising authority of the project and was kept unchanged throughout all experiments in order to ensure an objective evaluation under the official data partition.

As shown in Table 2, the dataset exhibits a pronounced class imbalance problem, with the ratio between UAV and Bird samples being approximately 1:11. This severe imbalance constitutes the main motivation for introducing the physics-constrained TimeGAN (PC-TimeGAN) data augmentation network in this study. PC-TimeGAN generated 7000 synthetic UAV trajectories in total. From these generated samples, 1000 trajectories were randomly selected and added only to the training set for data augmentation, while the test set remained unchanged and consisted entirely of real measured samples. As a result, the class ratio of UAV to Bird samples in the training set was improved from 1:11 to 1:4.8. A fully balanced ratio (e.g., 1:1) was not adopted, because introducing too many synthetic samples may lead to distribution shift and cause the model to fit statistical artifacts of generated data rather than the true physical motion patterns of real targets. In addition, during training, the generated samples were assigned a weight of 0.5, while real samples were assigned a weight of 1.0.

For each trajectory sample, the first five consecutively detected measurement points were extracted. After feature engineering, each sample was represented as a 15-dimensional feature vector. Among these features, eight dimensions were directly obtained from radar measurements, including azimuth angle, elevation angle, target range, target altitude, velocity magnitude, velocity direction angle, signal-to-noise ratio (SNR), and vertical velocity component. The remaining seven dimensions were kinematic derived features obtained through geometric calculations, including velocity variation, altitude variation, azimuth variation, horizontal velocity, flight path angle, jerk, and elevation-angle variation. These features were designed to comprehensively characterize the target’s maneuverability, motion continuity, and flight-pattern-related dynamic properties.

Figure 6 and Figure 7 present typical trajectories of birds and UAVs, respectively. The results show that UAVs exhibit motion patterns such as straight flight and circling, while birds also demonstrate similar movement characteristics, including straight flight, ascending motion, and circling. The high degree of similarity in the geometric trajectory patterns of the two target classes highlights the difficulty and challenge of UAV/bird classification in practical engineering applications.

#### 4.1.2. Experimental Settings

The proposed recognition network was implemented based on the PyTorch 2.2.0 with CUDA 12.1 framework and trained on an NVIDIA RTX 3090 GPU platform. Model optimization was performed using the AdamW optimizer with an initial learning rate of 0.001. In addition, a cosine annealing learning rate scheduler was adopted to dynamically adjust the learning rate during training. The main hyperparameter settings were as follows: the batch size was set to 64, and the maximum number of training epochs was set to 100. To prevent overfitting and improve training efficiency, an early stopping strategy was employed, such that training was automatically terminated if the training loss did not decrease further for 15 consecutive epochs.

The core model parameters and dataset partition settings are summarized as follows. The input trajectory length was set to five time steps, and the hidden dimension of the network was set to 64. In the multi-scale TCN front-end, each branch contained two TemporalBlocks, while the back-end Transformer encoder consisted of two stacked layers with four attention heads. In addition, an independent test set containing 611 samples was constructed. This test set was excluded from model training and was used exclusively to quantitatively evaluate the generalization capability of the proposed model.

#### 4.1.3. Evaluation Metrics

To comprehensively evaluate the performance of the proposed model in the UAV/bird classification task, UAV recall and the false alarm rate (FAR) of misclassifying birds as UAVs were adopted as the primary evaluation metrics. In addition, the classification results were quantitatively analyzed based on the confusion matrix. Let true positive, false positive, true negative, and false negative be denoted by TP, FP, TN, and FN, respectively, where TP represents the number of UAV samples correctly classified as UAVs, FP represents the number of bird samples incorrectly classified as UAVs, TN represents the number of bird samples correctly classified as birds, and FN represents the number of UAV samples incorrectly classified as birds.

The UAV recall is defined as

(20) $$\mathrm{Recall}=\frac{TP}{TP+FN},$$

which reflects the model’s ability to detect true UAV targets. A higher recall indicates a lower probability of missed UAV detections.

The false alarm rate of misclassifying birds as UAVs is defined as

(21) $$\mathrm{FAR}=\frac{FP}{FP+TN},$$

which reflects the probability that Bird targets are incorrectly identified as UAVs. A lower FAR indicates a stronger capability of the model to suppress confusing Bird targets such as birds.

The precision is defined as

(22) $$\mathrm{Precision}=\frac{TP}{TP+FP},$$

which reflects the proportion of predicted UAV samples that are truly UAVs. A higher precision indicates a lower probability of falsely identifying Bird targets as UAVs.

The F1-score is defined as

(23) $$F1=\frac{2\times \mathrm{Precision}\times \mathrm{Recall}}{\mathrm{Precision}+\mathrm{Recall}},$$

which provides a harmonic-mean evaluation of precision and recall, and is particularly useful for assessing classification performance under imbalanced-data conditions.

It should be noted that, in trajectory recognition tasks, there is usually a trade-off between recall and FAR. When the decision boundary is relaxed toward the UAV class, some UAV samples originally located near the decision boundary can be correctly recognized, thereby improving recall. However, at the same time, some bird samples with motion patterns similar to those of UAVs are also more likely to be misclassified as UAVs, resulting in an increased FAR. Conversely, when the decision boundary becomes more conservative, the FAR can be reduced, but more UAV samples may be missed, which in turn leads to a decrease in recall. Therefore, this study focuses particularly on the model’s ability to control the FAR under high-recall conditions.

### 4.2. Experimental Results and Analysis

To verify the effectiveness and superiority of the proposed method, comparative experiments were conducted on the dataset used in this study. Traditional machine learning methods, including Random Forest, SVM, and KNN, as well as deep learning models such as 1D-CNN, LSTM, GRU, and Transformer, were selected as baseline models. The quantitative results in terms of the two core metrics, namely recall and false alarm rate (FAR), are presented in Table 3.

As shown in Table 4, the proposed TCN-Transformer model contains 169.2 K trainable parameters, with a single-sample inference latency of 3.384 ms and a throughput of 75,535 FPS. Although it is not the fastest among all compared methods, its computational cost is even negligible for practical deployment, and the overall decision delay is mainly dominated by radar signal processing and data processing procedures rather than network inference.

As shown in Table 3, the overall performance of the traditional machine learning methods is unsatisfactory for this task. The UAV recall rates of Random Forest, SVM, and KNN are only 25.00%, 37.50%, and 35.00%, respectively. These results indicate that, under conditions where UAVs and birds exhibit similar motion patterns and the class distribution is highly imbalanced, traditional methods that rely mainly on shallow decision boundaries or static feature mappings are unable to adequately characterize the complex nonlinear temporal features embedded in low-altitude target trajectories, and therefore fail to achieve satisfactory recognition performance.

In contrast, the deep learning methods are able to automatically learn more discriminative temporal representations in an end-to-end manner and thus outperform the traditional machine learning methods in terms of UAV recall. After extending the relevant baselines to the full 15-dimensional feature set, it can be seen that richer input features improve the performance of several baseline models to some extent. Among them, the Transformer baseline with 15-dimensional input features achieves a UAV recall of 72.50% and an FAR of 3.50%, indicating that higher-order kinematic features are indeed beneficial for UAV/bird classification. Nevertheless, even under the same 15-dimensional input setting, the overall recall for UAV targets remains limited compared with the proposed method.

From the perspective of additional metrics for imbalanced classification, the proposed TCN-Transformer achieves a precision of 64.00% and the best F1-score of 0.7111 among all compared methods. Although its precision is not the highest, it attains the highest UAV recall of 80.00%, indicating a better overall balance between minority-class detection sensitivity and classification robustness.

It is worth noting that, when only the first five trajectory points are used for rapid recognition, there exists an inherent trade-off between recall and FAR. Specifically, when the model is more inclined to identify UAV samples located near the decision boundary, the recall can be improved accordingly; however, some bird samples with motion patterns similar to those of UAVs are also more likely to be misclassified as UAVs, which leads to an increase in FAR. Therefore, for rapid low-altitude target recognition tasks, model performance should be evaluated comprehensively by jointly considering both recall and FAR.

Under the same 15-dimensional input setting, the proposed method achieves a UAV recall of 80.00% while maintaining the FAR at a relatively low level of 3.15%. Its overall performance is therefore significantly superior to that of all baseline models. These results demonstrate that the proposed method can substantially improve UAV recall without causing a pronounced deterioration in the false alarm rate, thereby outperforming both traditional machine learning methods and conventional deep learning approaches in terms of comprehensive classification performance. This result suggests that the proposed method achieves a practically favorable balance between UAV recall and FAR under the current rapid-recognition setting.

### 4.3. Ablation Study

To verify the effectiveness of the core components and design strategies of the proposed model, a series of ablation experiments were conducted on the measured dataset. By progressively removing or replacing key modules, the contribution of each component to classification performance was quantitatively analyzed. The experimental results are presented in Table 5.

As shown in Table 5, the convolution kernel configuration of the TCN has a significant influence on the recognition performance of the model. Experiments 1 and 2 adopt a single convolution kernel for temporal modeling. Among them, the model with kernel = 3 achieves a recall of 72.50%, outperforming the model with kernel = 5, which achieves 67.50%. This indicates that, when only the first five trajectory points are used for rapid recognition, a medium-scale convolution kernel is more effective than a larger kernel in capturing early local dynamic features. However, a single-scale convolution is still unable to simultaneously account for motion patterns across different temporal scales, which results in an inherent limitation in overall performance.

After adopting the dual-scale convolution combination of kernel = 2 and kernel = 5 in Experiment 3, the recall increases to 75.00%, indicating that, compared with a single-scale convolution kernel, multi-scale temporal convolution can improve the model’s ability to characterize local dynamic variations to a certain extent. However, the FAR also rises to 3.80%, suggesting that this approach remains insufficient in suppressing some confusing bird targets.

In Experiment 4, after removing PC-TimeGAN, the model achieves a recall of 75.00% and an FAR of 3.68%. By contrast, after incorporating PC-TimeGAN, the complete proposed method further improves the recall to 80.00% while reducing the FAR to 3.15%. These results demonstrate that the proposed physics-constrained generative network not only alleviates the scarcity of minority-class UAV samples, but also improves the model’s discriminative ability on boundary samples, thereby increasing UAV detection performance while effectively reducing the false alarm rate. This further indicates that the combination of multi-scale TCN-Transformer-based global temporal modeling and PC-TimeGAN-based data augmentation exhibits strong complementary and synergistic effects. To further analyze the trade-off between recognition performance and decision speed under different trajectory window lengths, additional experiments were conducted using 5-point, 10-point, and 15-point input sequences, as summarized in Table 6. The results show that increasing the window length from 5 to 10 points improves the classification performance, with the recall rising from 80.00% to 83.78% and the F1-score increasing from 0.7111 to 0.7561. However, this gain is accompanied by a significantly longer decision delay, since the estimated total delay increases from 10 s to 20 s when the radar scan period is 2 s per frame. When the window length is further extended to 15 points, the performance does not continue to improve; instead, the recall decreases to 77.14% and the FAR rises to 4.48%. This may be attributed to the reduced number of valid trajectory samples available for training and testing, as well as the accumulation of redundant or noisy temporal information in longer sequence segments. Therefore, although the 10-point setting achieves the best classification performance, the 5-point setting provides a more favorable trade-off between recognition accuracy and decision speed for rapid low-altitude target warning scenarios.

## 5. Conclusions

To address the challenges of data scarcity and feature ambiguity in radar classification of low-slow-small targets in complex low-altitude environments, this paper proposes a few-shot deep learning method that integrates generative adversarial networks with temporal feature modeling. By introducing physical boundary constraints into the improved PC-TimeGAN architecture, the proposed method generates synthetic trajectory data with realistic physical properties, overcomes the limitation of conventional geometric transformations that tend to destroy temporal structure, and effectively alleviates the problem of insufficient training samples. In addition, the proposed multi-scale TCN-Transformer architecture, which combines local fine-grained perception with global dependency modeling, is capable of simultaneously capturing short-term local features and the overall temporal evolution trend of target trajectories. Furthermore, the weighted joint loss strategy based on Focal Loss and Triplet Loss strengthens the model’s optimization capability for hard samples and significantly improves UAV recall while enhancing feature separability.

Experimental results demonstrate that the proposed method can substantially improve the recall of UAV targets and achieve favorable overall recognition performance under a controllable false alarm rate, indicating strong potential for practical engineering applications. The findings of this study provide effective technical support for real-time-capable UAV/bird target classification by low-altitude surveillance radar in complex environments.

In future work, we will further validate the proposed method on larger and more diverse measured radar trajectory datasets and investigate its generalization ability through alternative partition strategies and cross-validation-based evaluation. We will also explore the extension of the current framework to more complex low-altitude target categories and further optimize the trade-off between recall and false alarm rate in practical deployment scenarios.

## Figures and Tables

**Table 1:** Parallel TCN Branches and Their Feature Characteristics.

TCN Branch	Kernel Size	Receptive Field	Captured Features
Short-Term TCN	kernel_size = 2	1–2 time steps	Target maneuverability and acceleration variation
Medium-Term TCN	kernel_size = 3	3–5 time steps	Motion pattern, target position, and target velocity
Long-Term TCN	kernel_size = 5	5–10 time steps	Overall motion pattern and latent target-type characteristics

**Table 2:** Statistical Summary of the Dataset Split.

Dataset	UAV	Bird	Total	UAV Ratio
Training set	788	8550	9338	8.4%
Test set	40	571	611	6.5%
Total	828	9121	9949	8.3%

**Table 3:** Results of the comparative experiments.

Algorithm	Input Features	Precision (%)	Recall (%)	F1-Score	FAR (%)	Type
Random Forest [33]	8-D	90.91	25.00	0.3922	0.18	ML
Random Forest [33]	15-D	90.91	50.00	0.6452	0.35	ML
SVM [34]	8-D	68.18	37.50	0.4839	1.23	ML
SVM [34]	15-D	64.52	50.00	0.5634	1.93	ML
KNN [35]	8-D	50.00	35.00	0.4118	2.45	ML
KNN [35]	15-D	61.11	55.00	0.5789	2.45	ML
LSTM [17]	8-D	62.50	50.00	0.5556	2.10	DL
LSTM [17]	15-D	53.85	70.00	0.6087	4.20	DL
Transformer [18]	8-D	52.63	50.00	0.5128	3.15	DL
Transformer [18]	15-D	59.18	72.50	0.6517	3.50	DL
GRU [36]	8-D	63.89	57.50	0.6053	2.28	DL
GRU [36]	15-D	60.87	70.00	0.6512	3.15	DL
1D-CNN [37]	8-D	67.74	52.50	0.5915	1.75	DL
1D-DNN [37]	15-D	59.52	65.00	0.6098	2.80	DL
TCN-Transformer	15-D	64.00	80.00	0.7111	3.15	Ours

Note: 8-D and 15-D denote 8-dimensional and 15-dimensional input features, respectively; ML = machine learning; DL = deep learning; Ours = proposed method.

**Table 4:** Efficiency comparison of different methods.

Model	Parameters (K)	Single-Sample Latency (ms)	Batch Latency (256, ms)	FPS
TCN-Transformer (Ours, 15-D)	169.2	3.384	3.39	75,535
LSTM (15-D)	56.2	0.216	0.23	1,104,746
GRU (15-D)	42.7	0.200	0.22	1,150,104
1D-CNN (15-D)	17.7	0.356	0.32	809,970
Transformer (15-D)	103.1	0.543	0.53	481,134
RF (15-D)	N/A	50.483	37.79	6775

Note: 15-D denotes 15-dimensional input features. All latency results are measured on an NVIDIA GPU with batch size 256, averaged over 200 repeated runs.

**Table 5:** Results of the Ablation Experiments.

Experimental Configuration	Recall (%)	FAR (%)
Experiment 1: Single-scale TCN (kernel = 3)	72.50	2.98
Experiment 2: Single-scale TCN (kernel = 5)	67.50	2.80
Experiment 3: Dual-scale TCN (kernel = 2, 5)	75.00	3.80
Experiment 4: Without PC-TimeGAN	75.00	3.68
Proposed method	80.00	3.15

**Table 6:** Trade-off between recognition performance and decision delay under different trajectory window lengths.

Window Length	Recall (%)	FAR (%)	F1-Score	Single-Sample Inference Latency (ms)	Estimated Total Delay (s)
5 points	80.00	3.15	0.7111	4.108	10
10 points	83.78	3.59	0.7561	3.481	20
15 points	77.14	4.48	0.7200	3.306	30

Note: The estimated total delay includes both trajectory acquisition time and model inference time. The acquisition time is calculated based on a radar scan period of 2 s per frame. Since the inference latency is at the millisecond level, the total delay is dominated by the trajectory acquisition process.

## Data Availability

Data are contained within the article.
